# Lyn regulates mucus secretion and MUC5AC via the STAT6 signaling pathway during allergic airway inflammation

**DOI:** 10.1038/srep42675

**Published:** 2017-02-16

**Authors:** Xiaoyun Wang, Yin Li, Deyu Luo, Xing Wang, Yun Zhang, Zhigang Liu, Nanshan Zhong, Min Wu, Guoping Li

**Affiliations:** 1 Inflammation & Allergic Diseases Research Unit, Affiliated Hospital of Southwest Medical University, Luzhou 646000, Sichuan, China; 2 The First Clinic College, Chongqing Medical University, Chongqing 401331, China; 3 State Key Laboratory of Respiratory Disease for Allergy at Shenzhen University, School of Medicine, Shenzhen University, Nanhai Ave 3688, Shenzhen Guangdong 518060, P.R. China; 4 State Key Laboratories of Respiratory Disease, Ghuangzhou Medical University, Guangdong 510120, P.R. China; 5 Department of Basic Biomedical Sciences, School of Medicine and Health Sciences, University of North Dakota, 501 N Columbia Rd, Grand Forks, ND 58203-9037, USA.

## Abstract

Hypersecretion of mucus is an important component of airway remodeling and contributes to the mucus plugs and airflow obstruction associated with severe asthma phenotypes. Lyn has been shown to down-regulate allergen-induced airway inflammation. However, the role of Lyn in mucin gene expression remains unresolved. In this study, we first demonstrate that Lyn overexpression decreased the mucus hypersecretion and levels of the *muc5ac* transcript in mice exposed to ovalbumin (OVA). Lyn overexpression also decreased the infiltration of inflammatory cells and the levels of IL-13 and IL-4 in OVA-challenged airways. Whereas Lyn knockdown increased the IL-4 or IL-13-induced MUC5AC transcript and protein levels in the human bronchial epithelial cell line, 16HBE, Lyn overexpression decreased IL-4- or IL-13-induced MUC5AC transcript and protein levels. Overexpression of Lyn also decreased the expression and phosphorylation of STAT6 in OVA-exposed mice, whereas Lyn knockdown increased STAT6 and MUC5AC levels in 16HBE cells. Finally, chromatin immunoprecipitation analysis confirmed that Lyn overexpression decreased the binding of STAT6 to the promoter region of *Muc5ac* in mice exposed to OVA. Collectively, these findings demonstrated that Lyn overexpression ameliorated airway mucus hypersecretion by down-regulating STAT6 and its binding to the MUC5AC promoter.

Mucous metaplasia is an important component of airway remodeling associated with severe asthma phenotypes. However, mucus dysfunction is often underappreciated by clinicians whose attention is primarily focused on reversing the bronchoconstriction and inflammation in asthma. Mucins are the major macromolecular component of the mucus^1^, and MUC5AC and MUC5B are the primary mucins in human airways. MUC5B is the principal gel-forming mucin in small airways under baseline conditions in humans and mice. MUC5B, but not MUC5AC, is essential for airway homeostasis and antibacterial defense^2^. MUC5AC is the principal gel-forming mucin that is up-regulated in airway inflammation^3^. Up-regulated production of MUC5AC contributes to mucous plugs and airflow obstruction in asthmatic patients^4^,^5^,^6^. Airflow limitation caused by mucus hypersecretion hampers the reversal of inflammation and the clearance of the excess secretions^7^.

The levels of T helper type 2 cytokines such as interleukin-4 (IL-4) and interleukin-13 (IL-13) are significantly increased during the pathogenesis of allergic asthma, and these cytokines induce mucus production in the airways^8^,^9^. IL-13 plays a critical role in asthmatic mucus overproduction. Glucocorticoids are not sufficient to suppress IL-13-induced goblet cell hyperplasia^10^. It has been shown that the phosphoinositide 3-kinase (PI3K)-nuclear factor of activated T cells (NFAT) pathway and STAT6/SAM domain-containing prostate-derived Ets factor (SPDEF) are involved in IL-13-induced MUC5AC expression^11^,^12^. STAT6 is an important transcription factor and is activated by IL-4 and IL-13 via the IL-4Ra subunit in their cognate receptors^13^. The IL-4Ra receptor and STAT6 play key roles in the IL-13-induced mucus production in mouse airway epithelial cells^14^. STAT6-knockout mice were protected from airway inflammation in the murine model of OVA-induced allergic airway inflammation^15^. However, it remains unclear whether STAT6 directly regulates the levels of MUC5AC or of other mucus regulators.

Lyn kinase, a member of the Src kinase family, is a non-receptor cytoplasmic tyrosine kinase that regulates various cellular processes and plays a crucial role in the immune response and inflammatory reactions. Lyn is associated with asthma due to its participation in IL-5 receptor signaling and Janas M. *et al*. also have reported that Lyn is a negative regulator of IL-4 signaling^16^,^17^,^18^,^19^. Lyn has been regarded as an activating regulator, but it exhibits both activating and inhibitory roles in different diseases or body’s conditions^20^,^21^,^22^. Lyn was necessary for the antiapoptotic effect of IL-5 in eosinophils. Suppressing Lyn kinase activity blocked the ability of IL-5 to prevent eosinophil death^18^. However, Lyn-deficient mice are prone to severe and persistent asthma, indicating that Lyn negatively regulates the progression of asthma^23^. However, the effect of Lyn overexpression in asthma remains unclear, including the molecular mechanisms by which Lyn modulates asthmatic pathology, especially airway mucus hypersecretion. Herein, we examined the regulatory role of Lyn knockdown and over-expression in mucous secretion using our recently created Lyn-transgenic mice and human airway epithelial cells. These findings demonstrated that Lyn overexpression ameliorated airway mucus hypersecretion via down-regulation of STAT6 as well as binding to the MUC5AC promoter.

## Methods

### Reagents

A Lyn-specific siRNA were purchased from Santa Cruz Biotechnology (Santa Cruz, CA). The antibodies for histology were purchased from Santa Cruz Biotechnology: MUC5AC (mucin 5AC, Clone # K-20; Cat # sc-16903), STAT6 (Clone # S-20, Cat # sc-621), β-actin (Clone # N-21, Cat # sc-130656), and phospho-STAT6 (Clone # Tyr641, Cat # sc-11762). The ELISA kits for IL-4, and IL-13 were purchased from Beijing Yonghui biotechnology Co. Ltd (Beijing, China). The MUC5AC-pGL3 luciferase vector was constructed by our laboratory based on the pGL3 luciferase reporter vector (Promega) following the manufacturer’s instructions.

### Immunization and challenge protocol

Sensitization of the Lyn-transgenic(Lyn^tg^) and wild type (WT) mice with OVA was performed using previously described methods^24^. The Lyn^tg^ and WT mice were sensitized by an intraperitoneal injection of 20 μg of OVA and 1 mg of aluminum hydroxide on days 0 and 14. The mice were challenged with an intranasal instillation of 10 μg of OVA in 50 μl of phosphate buffered saline (PBS) 5 times/week from week 3 to week 8 (for duration of 5 weeks). The Lyn^tg^ mice and WT mice in the control group were given PBS alone. The mice were sacrificed with an intraperitoneal injection of 40 mg/kg ketamine 24 hours after the final intranasal challenge. The lung tissues were stored in liquid nitrogen or fixed in 10% neutral-buffered formalin and embedded in paraffin. The Lyn-transgenic mice on a C57BL/6 J genetic background were generated by Cyagen Biosciences, Inc. (Guangzhou, China). The mice were backcrossed to a C57BL/6 background for at least two generations and maintained under pathogen-free conditions, and experiments were initiated when mice were 6 to 8 weeks of age. Genotyping was performed by PCR and Western blotting. The Lyn-transgenic mice and C57BL/6 mice were maintained under specific pathogen–free conditions in the Animal Experimental Center of Southwest Medical University. All animal experiments were performed in accordance with the guidelines of the Animal Experiments Center of Southwest Medical University. All experimental protocols were approved by the Ethics Committee in Affiliated Hospital of Southwest Medical University, including any relevant details.

### Histological assessment

The mouse tracheas were cannulated with a 20 gauge catheter, and the lungs were lavaged four times with 1.0 ml of PBS. The cells in the bronchoalveolar lavage (BAL) fluid were enumerated using a hemocytometer. The BAL fluid was centrifuged (500 × g for 5 minutes at 4 °C) to obtain the cells, which were fixed and stained as described previously^25^. Differential cell counts for 200 cells from each sample were performed in duplicate on coded slides. The lung tissues were fixed in 10% neutral-buffered formalin and embedded in paraffin. Sections (5 μm) of the lung specimens were stained with standard hematoxylin-eosin staining (H&E) methods and periodic acid–Schiff (PAS) reagent. The inflammation index in the H&E-stained slides was scored for the severity of the inflammatory cell infiltrates around airways and vessels using previously described methods^26^. The index was calculated by multiplying severity by extent, with a maximum possible score of 9.

### Lung tissue homogenization and cytokines assay

The lung tissues were crushed and homogenized in radioimmunoprecipitation assay (RIPA) buffer. The protein concentrations were quantified using an Epoch multi-volume spectrophotometer system (Biotech, USA). The cytokine levels (IL-4 and IL-13) were determined in triplicate in total lung lysates from each animal using ELISAs.

### Cell culture

The human airway epithelial cells (16HBE) were cultured in DMEM/F12 culture medium with 10% fetal calf serum (FCS) at 37 °C in 5% CO_2_. After the 16HBE cells reached 85% confluence in 24-well and 6-well plastic plates, the medium was replaced with serum-free DMEM/F12 culture medium. The cells were then treated with IL-4 or IL-13 in serum-free culture medium. Recombinant human IL-4 and IL-13 were purchased from Peprotech, Inc. (Rocky Hill, New Jersey, USA), and were dissolved in PBS with 0.1% BSA for use at a final concentration of 1 ng/ml.

### Lentiviral expression constructs and DNA plasmid constructs

To generate a Lyn expression construct, human Lyn cDNA was amplified and cloned into the pLV.ExBi.P/Puro-EF1α-IRES-eGFP/pLV.Des3d.P/Puro vector using the Gateway technology kit according to the manufacturer’s instructions (Life Technologies Corporation, Carlsbad, California, USA). The Lyn cDNA was amplified using the specific primers: Lyn sense: 5′-CAAGTTTGTACAAAAAAGCAGGCT-3′, Lyn antisense: 5′-CACTTTGTACAAGAAAGCTGG-3′. The vector constructs were confirmed by DNA sequencing. The pLV.ExBi.P/Puro-EF1α-IRES-eGFP/pLV.Des3d.P/Puro vectors were transfected into human embryo kidney cells (293 T cells) to create infectious lentiviral vector-containing particles.

### Viral infection, transfection, and luciferase assay

The medium was replaced with serum-free DMEM/F12 culture medium after the 16HBE cells reached 85% confluence in 24-well and 6-well plastic plates. The lentiviral vector expressing Lyn was used at 10^9^ particles per well in the 6-well plates and 10^8^ particles per well in the 24-well plates. Lyn expression was analyzed by western blotting and by immunofluorescence using an SP5 Leica confocal microscope (Leica, Germany). The 16HBE cells were transfected with 20 mM Lyn siRNA (Santa Cruz Biotechnology) with LipofectAmine 2000 according to the manufacturer’s instructions. For the luciferase assay, a 1.5-kb segment of the 5′ flanking region of the human MUC5AC gene (from −1300 to +48) was cloned into the pGL3-Basic Luciferase Vector (Promega, Madison, WI). A PLR-TK vector was used as a control plasmid to measure transfection efficiency. The luciferase activity was measured according to our previous report^24^.

### RNA isolation, RT-PCR and qRT-PCR

Total RNA was extracted with the TRIzol RNA Reagent (Invitrogen Life Technologies, Carlsbad, CA) as recommended by the manufacturer. The RNA was resuspended in 50 μl of nuclease-free water, and its concentration was determined using a Nanodrop instrument (Epoch, BioTek). The RNA samples were stored at −80 °C until use. cDNA was reverse transcribed from an equal amount of RNA (1.5 μg per reaction) using avian myeloblastosis virus reverse transcriptase with oligo (dT) as the primer. Routine reverse transcription-PCR (RT-PCR) and quantitative real-time PCR (qRT-PCR) were used in our study. The mRNA levels of muc1, muc2, muc3, muc4, muc5ac, muc5b and muc13 and ChIP assays were detected using RT-PCR. qRT-PCR analysis was performed using SYBR Advantage qPCR Premix (Clontech, USA). The muc5ac mRNA levels were analyzed using 2-ΔΔCq threshold method with the Light Cycler 480 Multiple Plate Analysis Software (Roche Diagnostics, USA). The primer sequences for RT-PCR were shown in Table 1.The primer sequence of Mus musculus Muc5ac for qRT-PCR were as follows: Forward primer: 5′-TCTACC ACTCCCTGCTTCT-3′; Reverse primer: 5′-TGACTAACCCTCTTGA CC A C-3′.

### Immunofluorescent staining and Western blotting

Immunohistochemical staining was performed on glass slides using standard histological methods. The cells were fixed with cold methanol and blocked at room temperature. The cells were incubated with MUC5AC antibodies (Santa Cruz Biotechnology). A mouse isotype serum replaced the primary Ab as a negative control. FITC-conjugated goat anti-mouse or TRITC-conjugated anti-mouse secondary Abs were used to probe the primary Abs. The lung tissues removed from mice were cut by freezing microtome and applied for immunofluorescent staining as above. For western blotting, the lung tissues and cells were homogenized in RIPA buffer with an optional protease inhibitor mixture (Roche or Fisher Scientific), and the protein concentrations were measured using a Nanodrop instrument (Epoch, BioTek). Lysates from each sample were separated in a 10% SDS polyacrylamide gel electrophoresis at 100 V for 2 hours and transferred to a microporous polyvinylidene difluoride (PVDF) membrane at 100 mA for 2 hours. Lyn, β-actin, STAT6, phospho-Lyn and phospho-STAT6 antibodies (1:1000) were used for the Western blots. HRP-conjugated secondary antibodies were used to react with the primary Abs, and the bands were visualized using the *Pierce ECL* Western Blotting *kit (Pierce Biotechnology, USA).* AlphaEaseFC software was used to quantify protein expression.

### Chromatin immunoprecipitation (ChIP) assay

ChIP assays were performed using EZ-Magna ChIP™ and One-Day Chromatin Immunoprecipitation kits (Millipore) according to previously described methods^27^. Frozen tissues were cut into 1–3 mm pieces. A volume of 1 ml of PBS with protease inhibitors (Roche, Germany) was added per 100 mg of tissue. The DNA-protein complexes were cross-linked and then sheared to 200–1000 bp using a sonicator. The sonicated nuclear fractions were divided for input control and incubation with a negative control IgG or the STAT6 (D3H4) rabbit mAb (Cell Signaling Technology, USA). The antibody protein-DNA complex was then pulled down with magnetic beads coupled to anti-mouse IgG. The pellets were washed with a series of wash buffers, and the protein-DNA complexes were eluted with 100 μl of ChIP Elution Buffer and 1 μl of Proteinase K. The DNA was purified using spin columns. Finally, the *Muc5ac* promoter region was amplified using RT-PCR and quantitative real-time PCR with primers specific for the STAT6-binding elements of the *MUC5AC* promoter region (from −879 to +1 bp): forward primer: 5′-CCATCCCA GCAGACATGAAA-3′, reverse primer: 5′-CTATTAACCTCCTGAGC AACCC-3′. The specificity of each primer was confirmed by analyzing the melt curve and the amplification plot.

### Statistical analysis

All data are presented as the *mean ± s.d*. The statistical analyses were performed using ANOVA. All the statistical analyses were conducted using SPSS 17.0 software. The level of significance was defined as a P value less than or equal to 0.05.

## Results

### Overexpression of Lyn attenuated OVA-induced mucus hypersecretion and muc5ac expression

**Figure 1:**
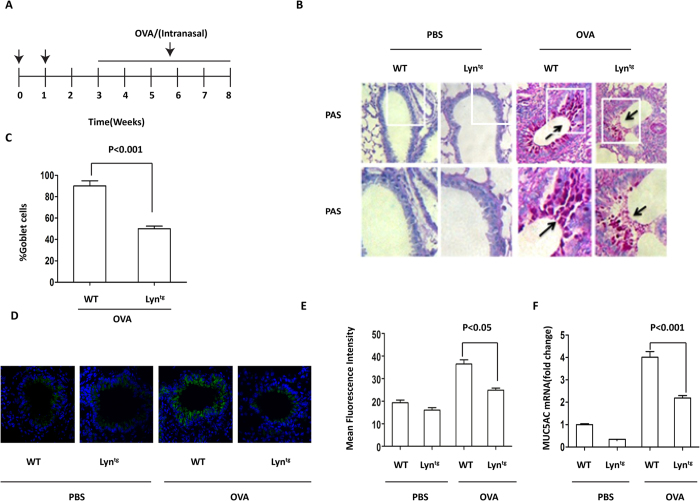
Mucus hypersecretion and *muc5ac* transcripts in OVA-induced Lyn^tg^ mice. (**A**) Protocol for sensitization and challenge of Lyn^tg^ mice and WT mice with OVA (n = 8 mice for each group). (**B**) PAS staining of epithelial goblet cells in the lungs of WT and Lyn^tg^ mice exposed to OVA or PBS (original magnification, x200). (**C**) The PAS-positive cell percentage was quantified in 10 random fields (original magnification, x200). (**D**) Muc5ac in 16HBE cells was determined by immunofluorescence (original magnification, x200, the blue color is for DAPI). (**E**) The intensity of muc5ac was quantified. (**F**) The mRNA expression of *muc5ac* was determined in the lungs of Lyn^tg^ mice and WT mice using qRT-PCR (n = 3 mice for each group).

Based on our previous findings showing mucus hypersecretion during exposure of Lyn^−/−^ (Lyn knockout) mice to HDM^24^, we investigated mucus secretion and *muc5ac* expression using a transgenic approach. To more thoroughly elucidate the critical role of Lyn in asthmatic pathology, we generated Lyn transgenic mice (Lyn^tg^ mice) and successfully verified the overexpression of Lyn in these mice by western-blot. We then backcrossed these mice with C57BL/6 J for 2 generations and challenged them as well as control mice with OVA. We employed an initial sensitization using an intraperitoneal (i.p.) injection of OVA followed by repeated intranasal instillations to induce chronic airway inflammation in the Lyn^tg^ and WT mice (Fig. 1A). We further confirmed that Lyn kinase regulates mucus secretion and *muc5ac* expression in a murine model of allergic airway inflammation. The lungs of the WT and Lyn^tg^ mice exposed to PBS remained normal, with few mucin-positive goblet cells and trace amounts of *muc5ac* (Fig. 1B,D,F). The lungs of the WT and Lyn^tg^ mice exposed to OVA (at 3, 6 and 8 weeks) showed an increased number of mucin-positive goblet cells (Fig. 1B,C; Supplementary Figure S1A). At 8 weeks, we observed a robust decrease in the number of mucin-positive goblet cells in the Lyn^tg^ mice exposed to OVA compared to the WT mice (2.0-fold decrease; Fig. 1C; *P* < 0.001). Immunofluorescent staining of lung tissue showed that expression of muc5ac in Lyn^tg^ mice exposed to OVA was less than WT mice exposed to OVA (Fig. 1D,E; Supplementary Figure S1B; *P* < 0.05. In Supplementary Figure S1B, “I&II” both refer to the negative control of immunohistochemistry staining in Fig. 1D. “I” indicates the sample’s autofluorescence. “II” shows non-specific fluorescence.). To examine the transcript levels of mucous secretion genes, RT-PCR was utilized to detect the mRNA levels of muc1, muc2, muc3, muc4, muc5ac, muc5b and muc13 in lung tissue. The transcript levels for muc1, muc2, muc3, muc4, muc5b and muc13 in lung tissue were unaltered, whereas the Muc5ac transcript was significantly increased in the WT mice exposed to OVA (Fig. 1F; Supplementary Figure S1C). We also observed a robust decrease in *muc5ac* transcript levels in the Lyn^tg^ mice exposed to OVA (at 8 weeks) compared with WT mice (Fig. 1F; *P* < 0.001). Taken together, these findings indicate that Lyn overexpression decreased mucus secretion and *muc5ac* transcript levels in mice exposed to OVA.

### Overexpression of Lyn attenuated OVA-induced airway inflammation

**Figure 2:**
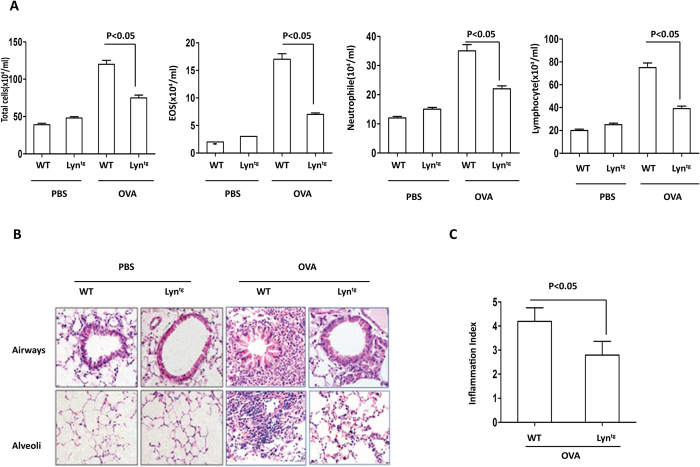
Airway inflammation in OVA-challenged Lyn^tg^ mice. (**A**) The total number of inflammatory cells as well as eosinophils, neutrophils and lymphocytes in the BAL of the WT and Lyn^tg^ mice were determined by differential cell analysis. (**B**) The lung tissues were stained using H&E (original magnification, x200). (**C**)The inflammatory cell infiltration index was determined in the lungs in (**B**) (10 random areas).

Having shown that the airway responds to inflammation with increased mucus secretion and that Lyn-deficiency enhanced the airway inflammation in mice exposed to HDM^7^, we analyzed the role of Lyn in regulating airway inflammation. The mice were challenged by intranasal instillations of 10 μg of OVA 5 times per week for 5 weeks. The total number of inflammatory cells as well as eosinophils, neutrophils and lymphocytes in the BAL had dramatic reduction in the OVA-treated Lyn^tg^ mice compared to the OVA-treated WT mice (Fig. 2A, *P* < 0.05). Inflammatory cell infiltration into the airways and alveoli was determined to be decreased in the OVA-treated Lyn^tg^ mice compared to the OVA-treated WT mice by H&E staining (Fig. 2B). The degree of cellular infiltration into the lungs was significantly decreased in the OVA-treated Lyn^tg^ mice compared to the OVA-treated WT mice (Fig. 2C, *P* < 0.05). These findings indicate that Lyn overexpression decreased the airway inflammation in mice exposed to OVA.

### Overexpression of Lyn decreased IL-13 and IL-4 during OVA-induced airway inflammation in mice

**Figure 3:**
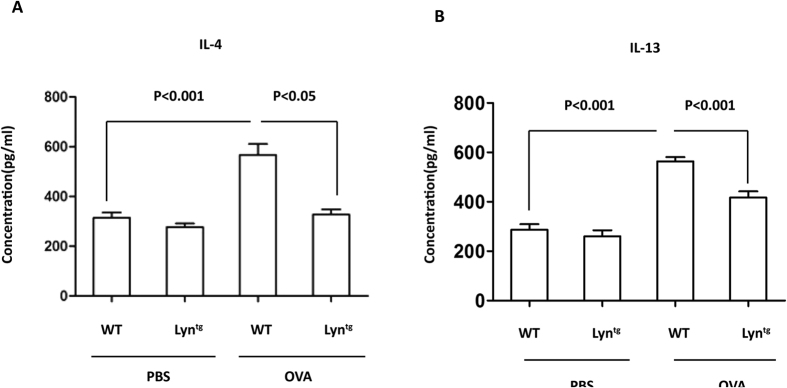
The levels of IL-13 and IL-4 in OVA-challenged Lyn^tg^ mice. The cytokine levels were measured by ELISA in triplicate samples of lung lysates from each animal. (**A**) IL-4 levels in lung tissue. (**B**) IL-13 levels in lung tissue (n = 3 mice for each group). The results are shown as the mean ± s.d.

Cytokines such as IL-13 and IL-4 induce goblet cell metaplasia and *muc5ac* gene expression^28^,^29^ Lyn transgenic mice were used to investigate the effect of Lyn on IL-13 and IL-4 in OVA-induced airway inflammation. There is a significant up-regulation of IL-4 and IL-13 when PBS and OVA treatment are compared in WT mice (Fig. 3A,B; *P* < 0.001). The levels of IL-4 and IL-13 in the lungs were significantly lower in the OVA-treated Lyn-transgenic mice compared to the OVA-treated WT mice (Fig. 3A,B; *P* < 0.05 or *P* < 0.001). These findings show that Lyn overexpression decreased IL-13 and IL-4 expression during OVA-induced airway inflammation.

### Lyn deficiency increased MUC5AC in IL-4/IL-13-exposed HBE cells

**Figure 4:**
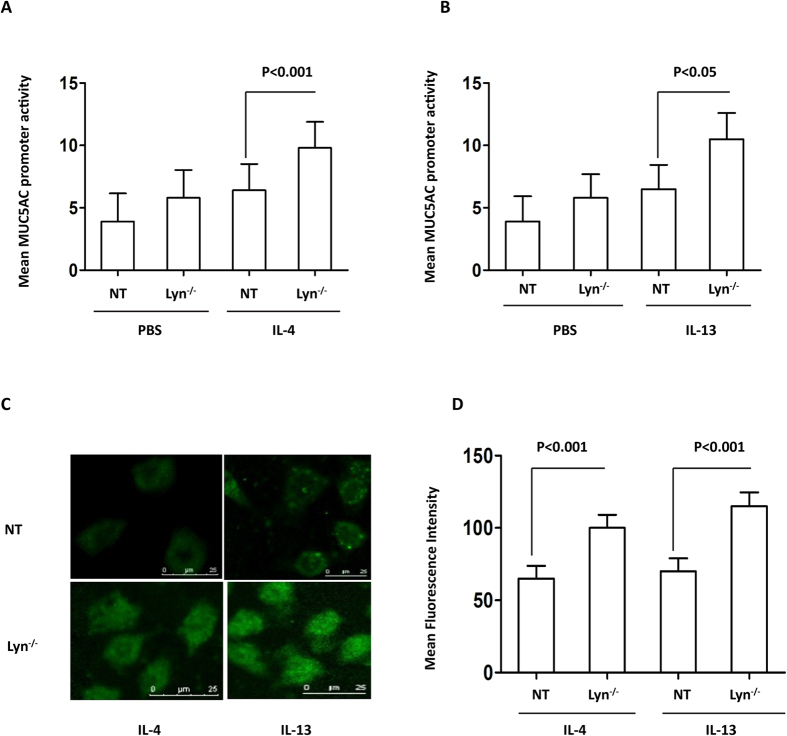
The levels of the MUC5AC transcript and protein in IL-4/IL-13-treated Lyn^−/−^ 16HBE cells *in vitro* (Lyn^−/−^ for siRNA treated cells, NT for untransfected cells). MUC5AC activation was determined using a luciferase promoter assay. The human airway epithelial 16HBE cells were cotransfected with the MUC5AC promoter-luciferase plasmid and Lyn siRNA. (**A**) The cells were then stimulated with 1 ng/ml IL-4 for 24 hours, and the luciferase activity was measured. (**B**) The cells were stimulated with 1 ng/ml IL-13 for 24 hours, and the luciferase activity was measured. (**C**) Immunohistochemical analysis of MUC5AC in Lyn^−/−^ cells and untransfected (NT) cells exposed to IL-4 or IL-13 for 24 hours. (**D**) Quantitation of the fluorescence intensity of MUC5AC per micrometer in 10 random fields as shown in (**C**). The results are shown as the mean ± s.d. All data are representative of three experiments, and a statistical analysis was performed.

To further evaluate the involvement of Lyn in IL-4- and IL-13-associated mucus secretion, MUC5AC and *muc5ac* expression was examined in IL-4- or IL-13-treated Lyn knockdown 16HBE cells using immunostaining and the MUC5AC PGL3 luciferase reporter assay. In cells transfected with the *MUC5AC* promoter construct, luciferase reporter gene activity increased in IL-4-treated Lyn knockdown 16HBE cells (Lyn^−/−^) compared to cells untransfected with LynsiRNA (NT cells) (Fig. 4A, *P* < 0.001). A similar phenomenon was also observed for IL-13-stimulated MUC5AC promoter luciferase activity in IL-13-treated Lyn knockdown 16HBE cells compared to NT 16HBE cells (Fig. 4B, *P* < 0.05). Control (scrambled) siRNA had been used in our previous study^24^. Thus, we have not used control siRNA this time (based on similar conditions). These results indicated that the region of the *MUC5AC* promoter spanning −1300/+48 contained the *cis*-acting element that was required for both IL-4- and IL-13-stimulated gene expression. The protein levels of MUC5AC were measured with MUC5AC antibody immunostaining. The level of MUC5AC in NT 16HBE cells increased after treatment with IL-4 or IL-13 for 24 hours compared to PBS treatment (Supplementary Figure S2A, S2B and S2C). The level of MUC5AC also increased ∼1.57-fold in IL-13-treated Lyn^−/−^cells and in IL-4 treated Lyn^−/−^ cells compared to NT cells (Fig. 4C,D, *P* < 0.001). Taken together, these studies suggest that Lyn regulates the transcription and translation of MUC5AC in airway epithelial cells exposed to IL-4 or IL-13. Lyn knockdown increased the IL-4 or IL-13-induced MUC5AC transcript and protein levels.

### Overexpression of Lyn decreased MUC5AC in IL-4/IL-13-treated HBE cells

**Figure 5:**
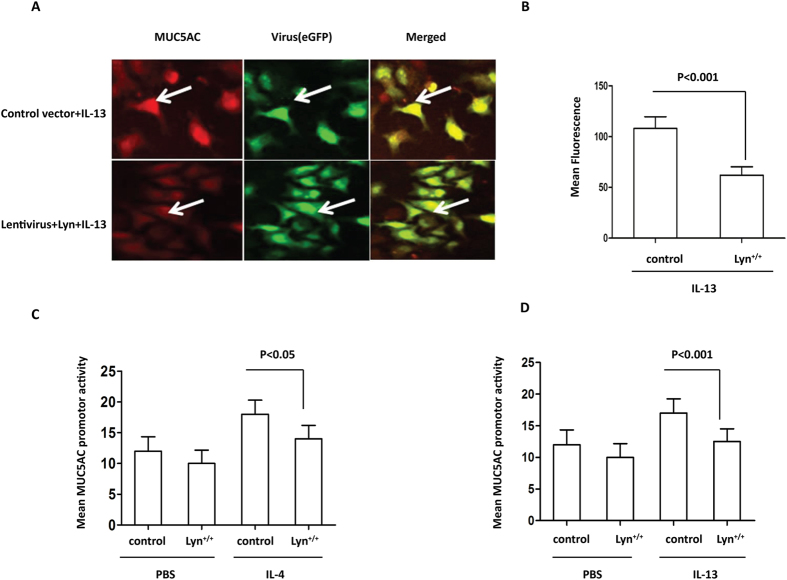
The levels of the MUC5AC transcript and protein in IL-4/IL-13-treated Lyn^+/+^.16HBE cells *in vitro*. MUC5AC activation was determined using a luciferase promoter assay. We infected 16HBE cells with the Lyn-eGFP-expression lentiviral vector and control vector respectively (10^8^ viral particles per well of a 24-well plate). (**A**) The cells were then stimulated with 1 ng/ml IL-13 for 24 hours. Immunohistochemical analysis of MUC5AC in Lyn^+/+^cells and control cells. The arrows indicate relevant markers in the positively stained cells: MUC5AC (red); eGFP or Lyn-eGFP (green). One representative experiment out of three is shown (original magnification x400). (**B**) Quantitation of the fluorescence intensity of MUC5AC per micrometer in 10 random fields of the images as shown in (**A**). (**C**) A MUC5AC promoter construct was transfected into control cells and Lyn^+/+^ cells. The cells were then stimulated with 1 ng/ml IL-4 for 24 hours. (**D**) The MUC5AC promoter construct was transfected into control cells and Lyn^+/+^ cells. The cells were then stimulated with 1 ng/ml IL-13 for 24 hours. The results are shown as the mean ± s.d. All data are representative of three experiments, and a statistical analysis was performed.

To further understand the role of Lyn, we assessed the MUC5AC levels in 16HBE cells using lentiviral Lyn-overexpression vectors. As shown in our previous studies, we generated Lyn-expression lentiviruses and infected 16HBE cells using 10^9^ viral particles per well of a 24-well plate. We next examined the effect of IL-13 on MUC5AC in cells infected with the Lyn-expressing lentivirus (Lyn^+/+^) by immunostaining. Lyn-infected or control vector-infected 16HBE cells were stimulated with IL-13 for 24 hours. We observed robust increases in the levels of MUC5AC in the IL-13-treated 16HBE cells transfected with control vector (control). However, Lyn-expression via lentiviral transduction suppressed the expression of MUC5AC in IL-13-treated 16HBE cells (Fig. 5A). MUC5AC showed a ∼1.7-fold decrease in mean fluorescence intensity in cells infected with the Lyn-expressing lentivirus and exposed to IL-13 compared to the corresponding control cells (Fig. 5B).

To further characterize the role of Lyn overexpression in regulating *muc5ac* transcription, we measured the luciferase activity in cultured 16HBE cells. In 16HBE cells transfected with the *muc5ac* promoter construct, the luciferase reporter gene activity decreased in IL-4-treated cells infected with the Lyn-expressing lentivirus compared to the corresponding control cells (Fig. 5C, *P* < 0.05). A similar result was also observed for the *muc5ac* promoter luciferase activity in IL-13-treated 16HBE cells infected with the Lyn-expressing lentivirus compared to those infected with the control vector (Fig. 5D, *P* < 0.001). These studies indicate that the levels of the *muc5ac* transcript and protein were clearly reduced in the Lyn*-*overexpressing human airway epithelial cells in response to IL-4/IL-13 treatment that simulated airway inflammation.

### STAT6 was critical for both IL-4- and IL-13-induced MUC5AC expression in Lyn- deficient 16HBE cells

**Figure 6:**
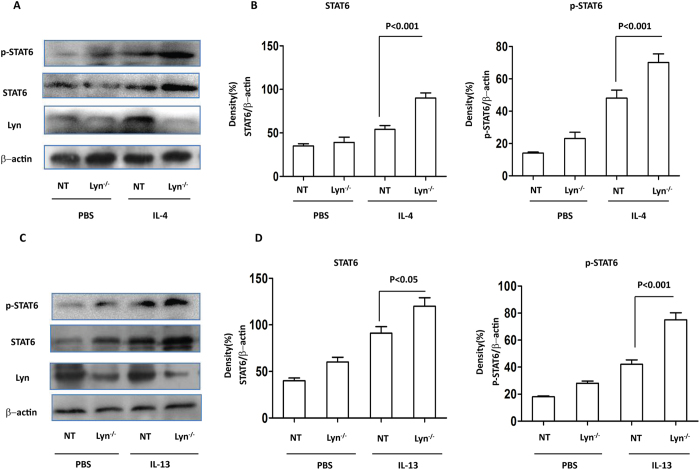
The expression and phosphorylation of STAT6 in IL-4/IL-13- challenged Lyn^/−^ 16HBE cells. The 16HBE cells were transfected with Lyn siRNA. (**A**) Western blot analysis of Lyn, STAT6 and the phosphorylation of STAT6 in Lyn^−/−^ and untransfected (NT) cells exposed to IL-4. (**B**) Relative density of STAT6 and phosphorylation of STAT6 in IL-4-treated Lyn knockdown and NT cells. (**C**) Western blot analysis of Lyn, STAT6 and phosphorylated STAT6 in Lyn^−/−^ and NT cells exposed to IL-13. (**D**) Relative density of STAT6 and phosphorylated STAT6 in IL-13-treated Lyn^−/−^ and NT cells. β-actin was used as the loading control. All data are representative of three experiments, and a statistical analysis was performed.

Because Lyn regulated MUC5AC in Lyn-transgenic mice and in IL-4/IL-13-treated cells, we next analyzed the transcriptional mechanism governing MUC5AC expression under these conditions. Previous studies have shown that STAT6 is critical to the development of allergen-induced AHR and mucus production^30^. IL-4 and/or IL-13 can initiate a cascade of STAT6-dependent signaling events in epithelial cells that lead to the formation of goblet cells^31^. The repression of STAT6 inhibits the IL-13-induced expression of MUC5AC^11^. However, the effect of STAT6 on MUC5AC remains undetermined. To further evaluate the involvement of STAT6 in IL-4/IL-13-induced MUC5AC expression, the expression and phosphorylation of STAT6 were studied in siRNA-mediated Lyn knockdown 16HBE cells (Lyn^−/−^) *in vitro* using Western blotting. IL-4 increased the protein expression and phosphorylation of STAT6 in Lyn knockdown 16HBE cells compared with untransfected (NT) 16HBE cells (Fig. 6A). The expression and phosphorylation of STAT6 increased ∼1.72-fold and ∼1.53-fold, respectively, in IL-4-treated cells compared to NT cells (Fig. 6B, *P* < 0.001). The expression and phosphorylation of STAT6 also increased by ∼1.22-fold and ∼1.75-fold in the IL-13-treated cells compared to the NT cells (Fig. 6C,D; *P* < 0.05 and *P* < 0.001, respectively). These results indicated that Lyn may play a key role in regulating the expression and phosphorylation of STAT6 to regulate MUC5AC.

To further determine whether the IL-4/IL-13-induced *MUC5AC* promoter activity was due to STAT6, the *muc5ac* transcript was measured in IL-4- or IL-13-treated STAT6 deficient 16HBE cells (STAT6^−/−^) using the MUC5AC PGL3 luciferase reporter vector *in vitro*. The knockdown of STAT6 by siRNA was shown in Sup Figure S3A. The luciferase reporter gene activity was significantly decreased ∼2.2-fold in IL-4-treated STAT6 knockdown 16HBE cells (STAT6^−/−^) compared to NT 16HBE cells (Supplementary Figure S3B). A similar observation was made for the IL-13-stimulated MUC5AC promoter luciferase activity in IL-13-treated STAT6 knockdown 16HBE cells compared to NT 16HBE cells. The luciferase reporter gene activity was significantly decreased (∼3.1-fold) in the IL-13-induced STAT6 knockdown 16HBE cells compared to the NT 16HBE cells (Supplementary Figure S3C). Taken together, these results indicated that STAT6 was critical for the IL-4/IL-13-induced MUC5AC expression. IL-4 and/or IL-13 can initiate a cascade of STAT6-dependent signaling events that culminate in MUC5AC expression.

### Overexpression of Lyn decreased STAT6 in IL-4/IL-13-treated 16HBE cells

**Figure 7:**
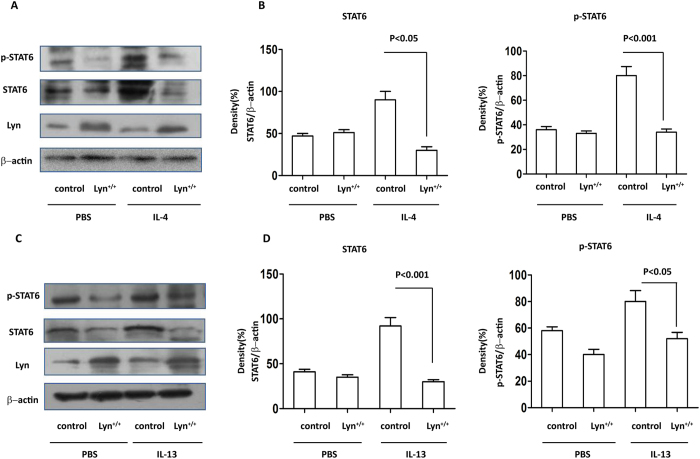
The expression and phosphorylation of STAT6 in IL-4/IL-13-treated Lyn^+/+^ 16HBE cells (Lyn^+/+^ for Lyn-expression lentiviral transfectd cells, control for control vector transfected cells). The 16HBE cells were infected with the Lyn-expression lentiviral vector and control vector respectively (10^9^ viral particles per well of a 6-well plate). (**A**) Western blot analysis of Lyn, STAT6 and phosphorylated STAT6 in Lyn^+/+^ and control cells after exposure to IL-4. (**B**) Relative density of STAT6 and phosphorylated STAT6 in IL-4-treated Lyn^+/+^ and control cells. (**C**) Western blot analysis of Lyn, STAT6 and phosphorylated STAT6 in Lyn^+/+^ and control cells after exposure to IL-13. (**D**) Relative density of STAT6 and phosphorylated STAT6 in IL-13-treated Lyn^+/+^ and control cells. β-actin was used as the loading control. All data are representative of three experiments, and a statistical analysis was performed.

The above studies implied that STAT6 is critical for both IL-4- and IL-13-induced MUC5AC expression in Lyn-deficient 16HBE cells *in vitro*. We next extended these studies to Lyn-overexpressing 16HBE cells (Lyn^+/+^) challenged with IL-4/IL-13. We infected 16HBE cells with the Lyn-expressing lentiviral vector and examined the effect of Lyn overexpression on STAT6 following IL-4/IL-13 challenge *in vitro*. The 16HBE cells infected with Lyn-expressing lentivirus were stimulated with IL-4/IL-13 for 24 hours. Figure 7A,B shows an approximately 2.96-fold decrease in STAT6 expression and an approximately 2.5-fold decrease in STAT6 phosphorylation in the IL-4-challenged Lyn^+/+^cells compared to the cells transfected with control vector (control). Furthermore, the expression of STAT6 also decreased approximately 3.4-fold in IL-13-challenged Lyn^+/+^cells compared to control cells. The phosphorylation of STAT6 decreased approximately 1.6-fold in the IL-13-treated Lyn^+/+^cells compared to the control cells (Fig. 7C,D). Collectively, these studies suggest that Lyn overexpression attenuated MUC5AC expression via negative regulation of the expression and phosphorylation of STAT6 in IL-4/IL-13-treated cells.

### Overexpression of Lyn attenuated STAT6 expression and STAT6 binding to the MUC5AC promoter in OVA-induced airway inflammation in mice

**Figure 8:**
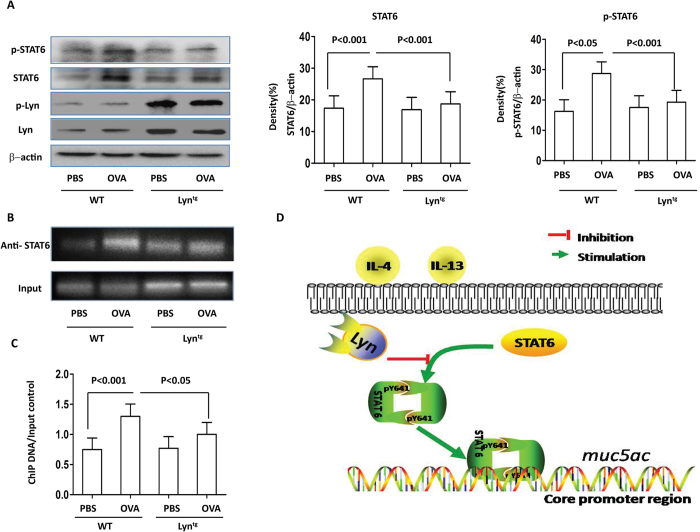
STAT6 and STAT6 binding to MUC5AC promoter during OVA-induced airway inflammation in mice. (**A**) Western blot analysis of Lyn, STAT6, phosphorylated STAT6 and phosphorylated Lyn in OVA-exposed Lyn^tg^ and WT mice. Relative density of STAT6 and phosphorylated STAT6 in lung tissue. (**B**) ChIP assays for STAT6 binding to the *muc5ac* promoter region in the lungs of OVA-challenged mice. Cross-linked chromatin was isolated and fragmented to 200–1000 bp. Anti-STAT6 Ab or control IgG was used to precipitate the DNA fragments bound to STAT6 or nonspecific proteins. The precipitation of the protein and DNA was measured by RT-PCR analysis with primers targeting the MUC5AC promoter regions. (**C**) Relative density of ChIP DNA/input control (in (**B**)) in lung tissue. (**D**) Diagram showing the cell signaling pathway associated with Lyn kinase.

Mice lacking STAT6 are protected from all of the pulmonary effects of IL-13. Reconstitution of STAT6 in epithelial cells only was sufficient for IL-13-induced mucus production^9^. Our previous studies established that the expression and phosphorylation of STAT6 increases in HDM-exposed Lyn^−/−^ mice^24^. We next investigated the expression and phosphorylation of STAT6 in OVA-exposed Lyn^tg^ mice. In our present study, the expression and phosphorylation of STAT6 in the lungs were decreased in OVA-exposed Lyn^tg^ mice compared to WT mice. Furthermore, the expression and phosphorylation of STAT6 decreased by approximately 1.44-fold and approximately 1.52-fold, respectively, in OVA-exposed Lyn^tg^ mice compared to OVA-exposed WT mice (Fig. 8A). Our studies provide the first evidence that overexpression of Lyn decreased the expression and phosphorylation of STAT6 during OVA-induced allergic airway inflammation in mice.

It is unknown whether STAT6 regulates MUC5AC via direct interaction between STAT6 and the *muc5ac* promoter. To determine whether *muc5ac* promoter activity is due to a direct interaction between STAT6 and the *muc5ac* promoter, ChIP assays were conducted on the lung tissue from OVA-induced allergic mice. The STAT6-binding elements of the *muc5ac* promoter region were amplified by RT-PCR. The nuclear fractions were incubated with a negative control IgG (Supplementary Figure S4) We found that precipitation using STAT6 Abs in the lung tissue from the OVA-challenged WT mice yielded a high PCR DNA band/input DNA ratio compared to that from the PBS control mice (Fig. 8B). In contrast, the PCR DNA band/input DNA ratio was approximately 1.34-fold lower in the OVA-exposed Lyn^tg^ mice compared to the OVA-exposed WT mice (Fig. 8C).

To further confirm these results, the relative gene expression data were analyzed using quantitative real-time PCR and the 2^-△△CT^ method. Our studies found that the fold enrichment of muc5ac expression increased approximately 5.9-fold in the OVA-exposed WT mice compared to the PBS-treated control mice (Supplementary Figure S5). Furthermore, applied bioinformatics for the identification of regulatory elements was performed using Jaspar (http://jaspar.genereg.net/) software. We found that the *MUC5AC* promoter activity may be due to a direct interaction between STAT6 and the *MUC5AC* promoter in the region flanking the STAT6 binding site (−879 ~ +1 bp). Taken together, these studies identified a novel pathway by which Lyn affected mucus hypersecretion by regulating STAT6. This is the first demonstration of STAT6 binding to the MUC5AC promoter in allergen-induced MUC5AC, *muc5ac* expression and the key role of Lyn in regulating STAT6 (Fig. 8D).

## Discussion

Mucus is an important component of both the physiological and pathological processes in airways^32^. A total of 20 human mucin (MUC) genes have been identified. However, their relative contributions to the development of asthma remain unclear^33^. In the present study, we demonstrated that the transcript levels for *muc1, muc2, muc3, muc4, muc5b* and *muc13* in lung tissue were unaltered, whereas *muc5ac* transcript expression was significantly increased during allergen-induced airway inflammation in mice (Supplementary Figure S1C). These results indicated that *muc5ac* expression plays a key role in airway inflammation and goblet cell metaplasia after antigen challenge. Mucus hypersecretion is induced in a time-dependent manner in mouse models of chronic airway inflammation. The number of mucin-positive goblet cells at 8 weeks was greater than that at 3 weeks (Fig. 1B and Supplementary Figure S1A). Although Lyn kinase has been implicated in the pathogenesis of asthma, only a few studies have addressed the role of Lyn in mucus secretion. In our previous studies^18^,^34^,^35^, Lyn-deficient mice exposed to house dust mite allergen exhibited mucus hypersecretion and increased *muc5ac* mRNA and MUC5AC protein expression^24^. The present study has carefully elaborated a novel and key role for Lyn in the regulation of airway mucin production. Lyn overexpression decreased mucus secretion and *muc5ac* transcription in mice exposed to allergens.

The pathology of asthma is characterized by structural changes, including goblet cell metaplasia and an increase in epithelial mucin stores^18^. The formation of pathological intraluminal mucus results in airway narrowing during asthma exacerbations. Goblet cell metaplasia results from chronic airway inflammation^36^. Previous studies have demonstrated that Lyn deficiency enhances the airway inflammatory response^24^. In the present study, the overexpression of Lyn attenuated airway inflammation and decreased total inflammatory cells including eosinophils, neutrophils and lymphocytes in the BAL as well as the degree of cellular infiltration in the lungs. The decreased airway inflammation was associated with lower levels of IL-13 and IL-4 in the Lyn transgenic mice exposed to allergen (Fig. 3). Our findings further confirmed that the Lyn-mediated decrease of IL-13 and IL-4 may contribute to lowered airway inflammatory responses.

T-helper type 2 (Th2) inflammation, mediated by IL-4, IL-5 and IL-13, is considered the central molecular mechanism underlying asthma^37^. IL-13 induces goblet cell metaplasia via forkhead box protein A2 (FoxA2), FoxA3 and SAM-pointed domain-containing ETS transcription factor (SPDEF). IL-17A induces mucus production and MUC5AC in a mechanism involving NFκB-mediated transcription, and NFκB specifically binds to position-3594/−3582 in the promoter of MUC5AC^38^,^39^. No previous study had linked Lyn kinase to the regulation of airway mucin production by IL-4/IL-13, but available databases led us to consider this possibility. We evaluated the relevance of Lyn-regulated *muc5ac* transcription in Lyn-knockdown cells and Lyn-overexpressing cells. Using a reporter-based promoter study, we demonstrated that Lyn indeed regulates the *muc5ac* transcript *in vitro.* Lyn deficiency increased the levels of the *muc5ac* transcript. In contrast, the overexpression of Lyn suppressed the IL-4/IL-13-induced transcription of the *muc5ac* gene. This is the first report describing a crucial role of Lyn in the transcriptional regulation of airway MUC5AC and *muc5ac* expression in response to IL-4 and IL-13.

IL-13 is a mediator of mucus hyperplasia. Therefore, IL-13 is an attractive drug target for treating asthma^40^. STAT6 plays a key role in affecting the transcription of many IL-4/IL-13-dependent genes^41^. Lyn influences the phosphorylation of various signaling molecules and transcription factors, including the STAT proteins^42^,^43^. Previous studies showed that Lyn interacted with STAT5 and directly activated STAT5. Maverick Lau *et al*. reported that genetic deletion of STAT6 in lupus-prone Lyn-deficient mice promotes autoimmune disease^44^. However, whether Lyn interacts with STAT6 or regulates STAT6 remains unclear. In the present study, using Lyn siRNA, we found that knocking down Lyn substantially increased the expression and phosphorylation of STAT6 in IL-4- and IL-13-treated 16HBE cells. However, using a Lyn-overexpressing lentiviral vector, we demonstrated that Lyn overexpression significantly decreased the expression and phosphorylation of STAT6 in the IL-4- and IL-13-treated 16HBE cells. These results indicated that Lyn exhibited a dual regulatory role regarding IL-4/IL-13-induced STAT6 activity.

Muc5ac is elevated by IL-13 via a STAT6-dependent pathway in mouse lungs^45^, and previous studies strongly support a pivotal role for IL-4 or IL-13 and STAT6 activation in mucus hypersecretion and plugging in the airways^30^. Using siRNA targeting STAT6 in our present studies, STAT6 knockdown strongly decreased the IL-4/IL-13-induced *Muc5ac* promoter activity (Supplementary Figure S3). Moreover, considering the similar findings in the Lyn knockout mice exposed to allergen, our data suggest that the downregulation of mucin production resulting from a decrease in STAT6 phosphorylation is dependent on Lyn overexpression in Lyn transgenic mice exposed to allergen (Fig. 8A). Thus, our data suggest that STAT6 is strongly implicated in IL-4/IL-13-mediated mucin production *in vitro*.

However, the sequence analysis of the Muc5ac 5′ flanking region showed no consensus motif for STAT6 (5′-TTCN4GAA-3′)^33^. The metaplasia airway mucous cells are incompletely dependent on the JAK/STAT6 pathway^45^. Previous results have found that SPDEF plays a critical role in mediating IL-13-induced MUC5AC synthesis in a manner dependent on STAT6. Phosphorylation of p38 MAPK is involved in IL-13-induced mucus cell metaplasia^46^. The IL-13-induced mucin expression may be associated with TGF-β2 up-regulation^47^. These results implicate a different gene regulatory mechanism. Sequence analysis of the Muc5ac promoter region was performed using applied bioinformatics software (http://jaspar.genereg.net/). The *Muc5ac* promoter in the region contains two STAT6 binding site motifs (−879 ∼ +1 bp). Using ChIP analysis of tissue from WT and Lyn transgenic mice exposed to allergen, we report for the first time that Lyn plays a crucial role in the transcriptional regulation of MUC5AC expression through STAT6 and the identified STAT6 response element locates in the promoter of the MUC5AC gene *in vivo*.

In summary, we have found that overexpression of Lyn attenuates OVA-induced mucus hypersecretion, *muc5ac* expression and airway inflammation. We further showed that Lyn kinase regulates the expression and phosphorylation of STAT6, which is associated with MUC5AC and *muc5ac* expression in allergen-induced mice and IL-4/IL-13-treated cells. We have further identified a critical STAT6 binding site in the *muc5ac* promoter essential for gene induction in OVA-induced mucus hypersecretion in mice. This study indicates that Lyn kinase plays a central role in mucus hypersecretion in antigen-challenged mice. Lyn regulated STAT6, which was associated with the development and progression of mucus hypersecretion and MUC5AC expression *in vivo* and *in vitro*.

## Additional Information

**How to cite this article:** Wang, X. *et al*. Lyn regulates mucus secretion and MUC5AC via the STAT6 signaling pathway during allergic airway inflammation. *Sci. Rep.* **7**, 42675; doi: 10.1038/srep42675 (2017).

**Publisher's note:** Springer Nature remains neutral with regard to jurisdictional claims in published maps and institutional affiliations.

## Supplementary Material

Supplementary Information

## Figures and Tables

**Table 1:** The primer sequences for RT- PCR.

Gene	Primer sequences	
Mus musculus mucin 1 (Muc1)	sense	5′-TCCAACTACTACCAAGAACTGAA-3′
	antisense	5′-CAAGGAAATAGACGATAGCCAA-3′
Mus musculus mucin 2 (Muc2)	sense	5′-ACCTGAAGAAATGTGTCACTGGG-3′
	antisense	5′-GTGGTAATGGTGGTAGAGATGGG-3′
Mus musculus mucin 3 (Muc3)	sense	5′-CTGGTGGAGAGCGTAGAGATAGA-3′
	antisense	5′-TTGGTGGCAGTGGAGTTGAAA-3′
Mus musculus mucin 4 (Muc4)	sense	5′-AGTTTCACTCCCACCATCTCTAT-3′
	antisense	5′-TTCTCCACTCCTCTTCTGCCT-3′
Muc5ac	sense	5′-GCCAAGTGCCAAAAGCAGTAGAG-3
	antisense	5′-GACCTGGGGTGTGGGTAGAAGA-3
Muc5b	sense	5′-CCATCCTCTGGGCTGAGTTGCTT-3′
	antisense	5′-TTGTGTTCTCGTCGGTCGCTTTC-3′
Muc6	sense	5′-GGCGTGTGTGTGGACTGGAGAA-3′
	antisense	5′-GAGGATGGGGCTGAAGGTGGT-3′
Muc13	sense	5′-CATCCTCATCTTGCTGATTGCTTTT-3′
	antisense	5′-TCTGCCCATTTCTCCTTGTCCT-3′
Muc19	sense	5′-CATTGGAGAGAAAGGAAAGTGTG-3′
	antisense	5′-GCTTGCATGTACGAAGAGGAT-3′
GAPDH	sense	5′-TCAACGGCACAGTCAAGG-3′
	antisense	5′-ACCAGTGGATGCAGGGA-3′
