# Supplementary material for: Lyn regulates mucus secretion and MUC5AC via the STAT6 signaling pathway during allergic airway inflammation
Source: Sci Rep. 2017 Feb 16;7:42675. doi: 10.1038/srep42675 (PMC5312001; doi:10.1038/srep42675)
Supplement: Supplementary Information [file srep42675-s1.pdf]

Supplementary information

Lyn regulates mucus secretion and MUC5AC via the STAT6 signaling pathway during allergic airway inflammation

Xiaoyun Wang [1]*, Yin Li[2]*, Deyu Luo[1], Xing Wang[1], Yun Zhang[1], Zhigang Liu[3], Nanshan Zhong[4], Min Wu[5,#], and Guoping Li[1#]

[1] Inflammation & Allergic Diseases Research Unit, Affiliated Hospital of Southwest Medical University, Luzhou 646000, Sichuan, China

[2] The First Clinic College, Chongqing Medical University, Chongqing 401331, China

[3] State Key Laboratory of Respiratory Disease for Allergy at Shenzhen University, School of Medicine, Shenzhen University, Nanhai Ave 3688, Shenzhen Guangdong 518060, PR China

[4] State Key Laboratories of Respiratory Disease, Ghuangzhou Medical University, Guangdong 510120, PR China

[5] Department of Basic Biomedical Sciences, School of Medicine and Health Sciences, University of North Dakota, 501 N Columbia Rd, Grand Forks, ND 58203-9037

*These authors contributed equally to this work.

[#] Corresponding authors: Guoping Li, E-mail: lzlgp@163.com, Tel: 868303165324, Fax: 868303165324. Min Wu, E-mail: min.wu@med.und.edu, Tel: 701 777-4875, Fax: 701 777-2382.

Running Title: **Lyn kinase regulates mucus hypersecretion in asthma**

Supplementary Figure S1. **Mucus hypersecretion and mucin transcripts in OVA-challenged Lyn^{tg} mice.** (A) PAS staining of the epithelial goblet cells in the lungs of WT and Lyn^{tg} mice after exposure to OVA or PBS at 3 and 6 weeks (original magnification, ×200). (B) Muc5ac in 16HBE cells was determined by immunofluorescence. A mouse isotype serum replaced the primary Ab as a negative control. "I" indicates sample autofluorescence. "II" shows non-specific fluorescence.
(C) Total RNA in the lungs was isolated, and the transcriptional changes were analyzed by RT-PCR. GAPDH was measured as an internal control.

Supplementary Figure S2. **The levels of the MUC5AC in PBS-treated Lyn$^{-/-}$ 16HBE cells in vitro (Lyn-/- for siRNA treated cells, NT for untransfected cells.).** (A) Immunohistochemical analysis of MUC5AC in Lyn$^{-/-}$ cells and untransfected (NT) cells exposed to PBS for 24 hours. (B) Mean fluorescence intensity of MUC5AC in Lyn$^{-/-}$ cells and NT cells exposed to PBS and IL-4. (C) Mean fluorescence intensity of MUC5AC in Lyn$^{-/-}$ cells and NT cells exposed to PBS and IL-13.

Supplementary Figure S3. ***Muc5ac* promoter activity in IL-4/IL-13-exposed STAT6$^{-/-}$ 16HBE cells(STAT6$^{-/-}$ for siRNA treated cells, NT for untransfected cells.).** MUC5AC activation was determined using a luciferase promoter assay. The human airway epithelial 16HBE cells were cotransfected with MUC5AC promoter-luciferase plasmid and STAT6 siRNA. (A) Western blot analysis of STAT6 in STAT6$^{-/-}$ and NT cells. (B) The cells were then stimulated with 1 ng/ml IL-4 for 24 hours, and the luciferase activity was measured. （C）The cells were stimulated with 1 ng/ml IL-13 for 24 hours, and the luciferase activity was measured. All data are representative of three experiments, and a statistical analysis was performed.

Supplementary Figure S4. **The negative control/anti-IgG control for the CHIP analysis.** The lung tissue was from WT and Lyn^{tg} mice treated with PBS and OVA. The sonicated nuclear fractions were divided for input control and incubation with a negative control IgG rabbit mAb.

Supplementary Figure S5. **STAT6 binding to the MUC5AC promoter in OVA-challenged mice.** ChIP assays for STAT6 binding to the *muc5ac* promoter region in the lungs of OVA-challenged WT mice. the MUC5AC promoter region was amplified using quantitative real-time PCR with primers specific for the STAT6-binding elements of the MUC5AC promoter region.

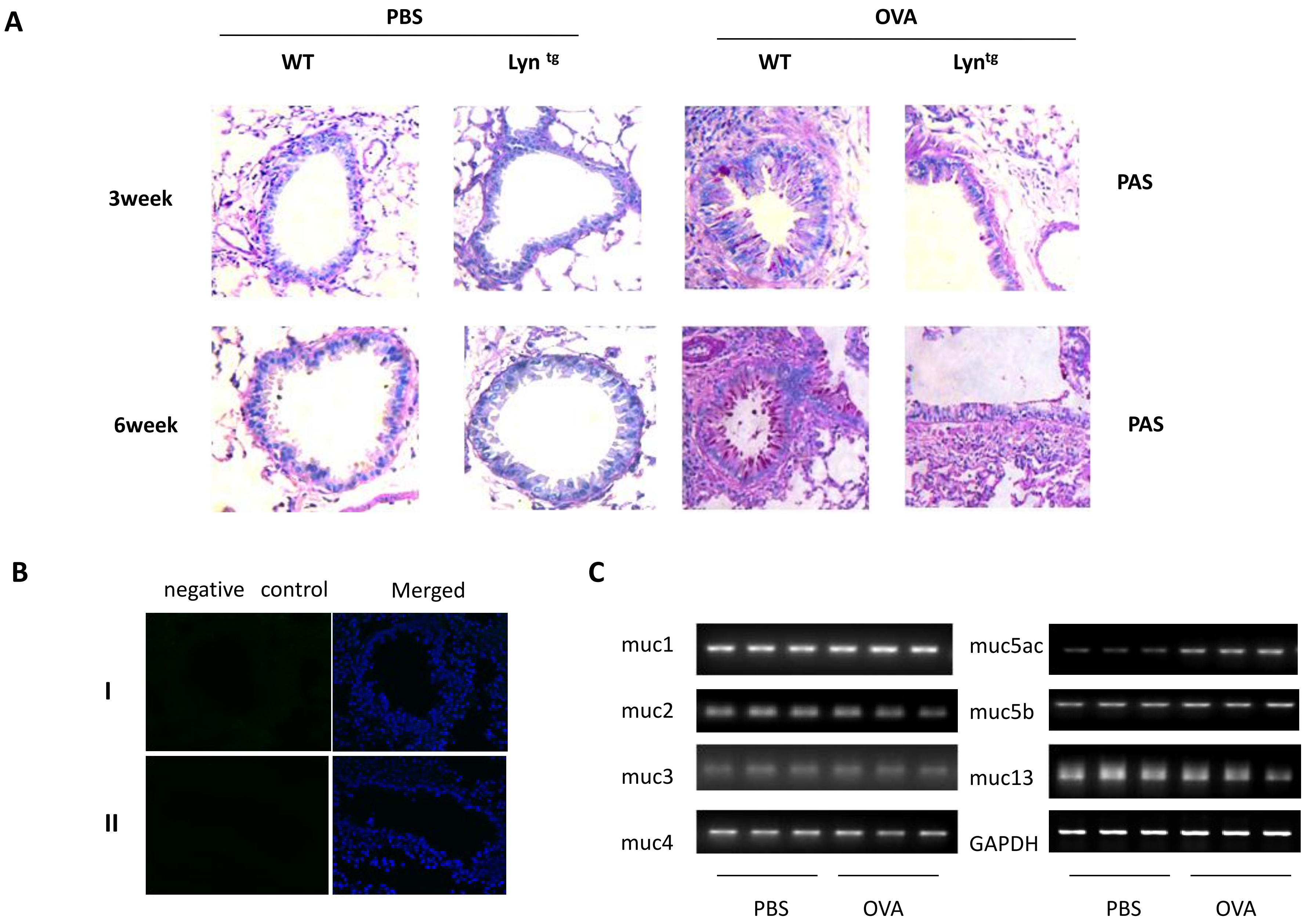

Sup Figure S1

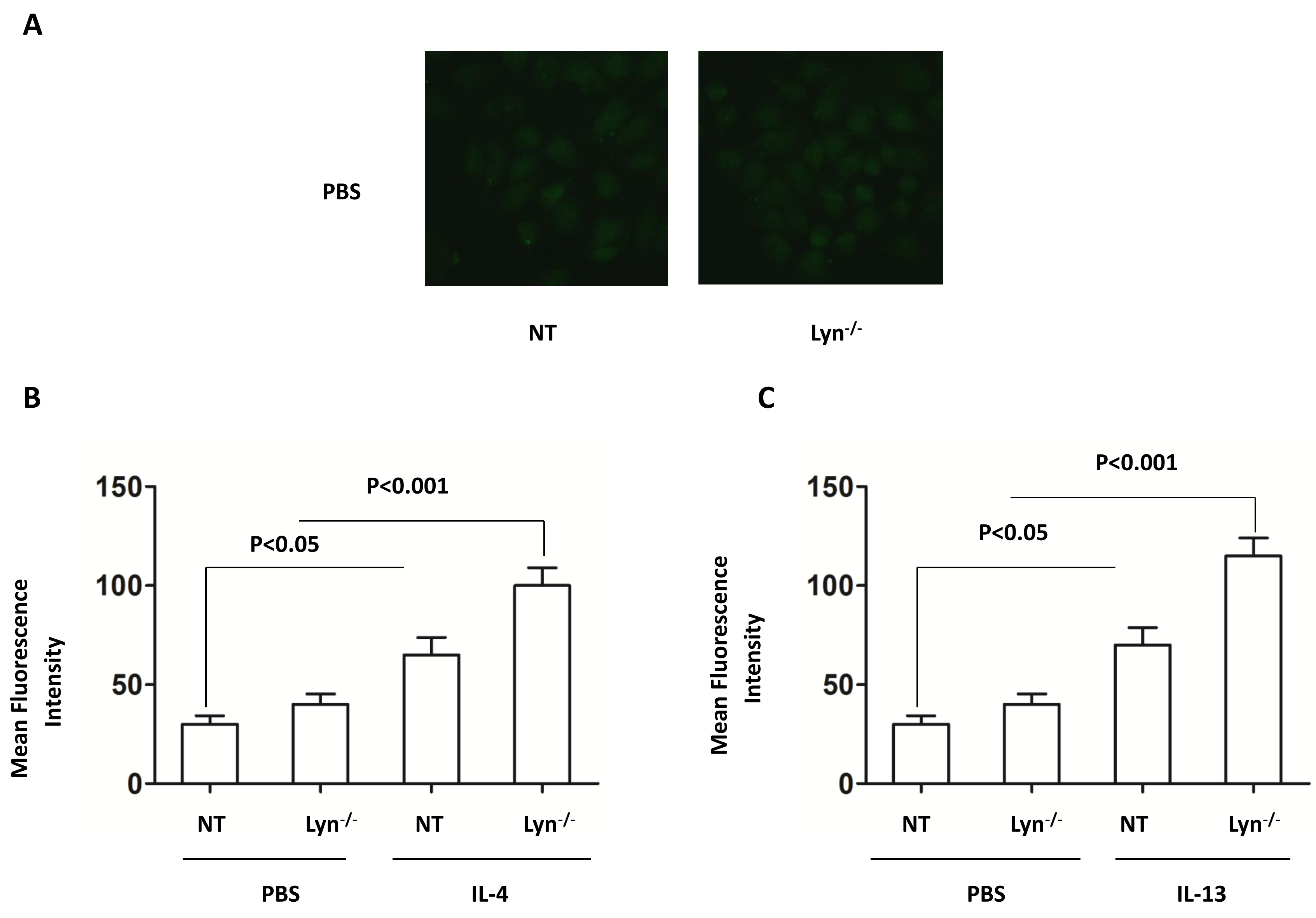

Sup Figure S2

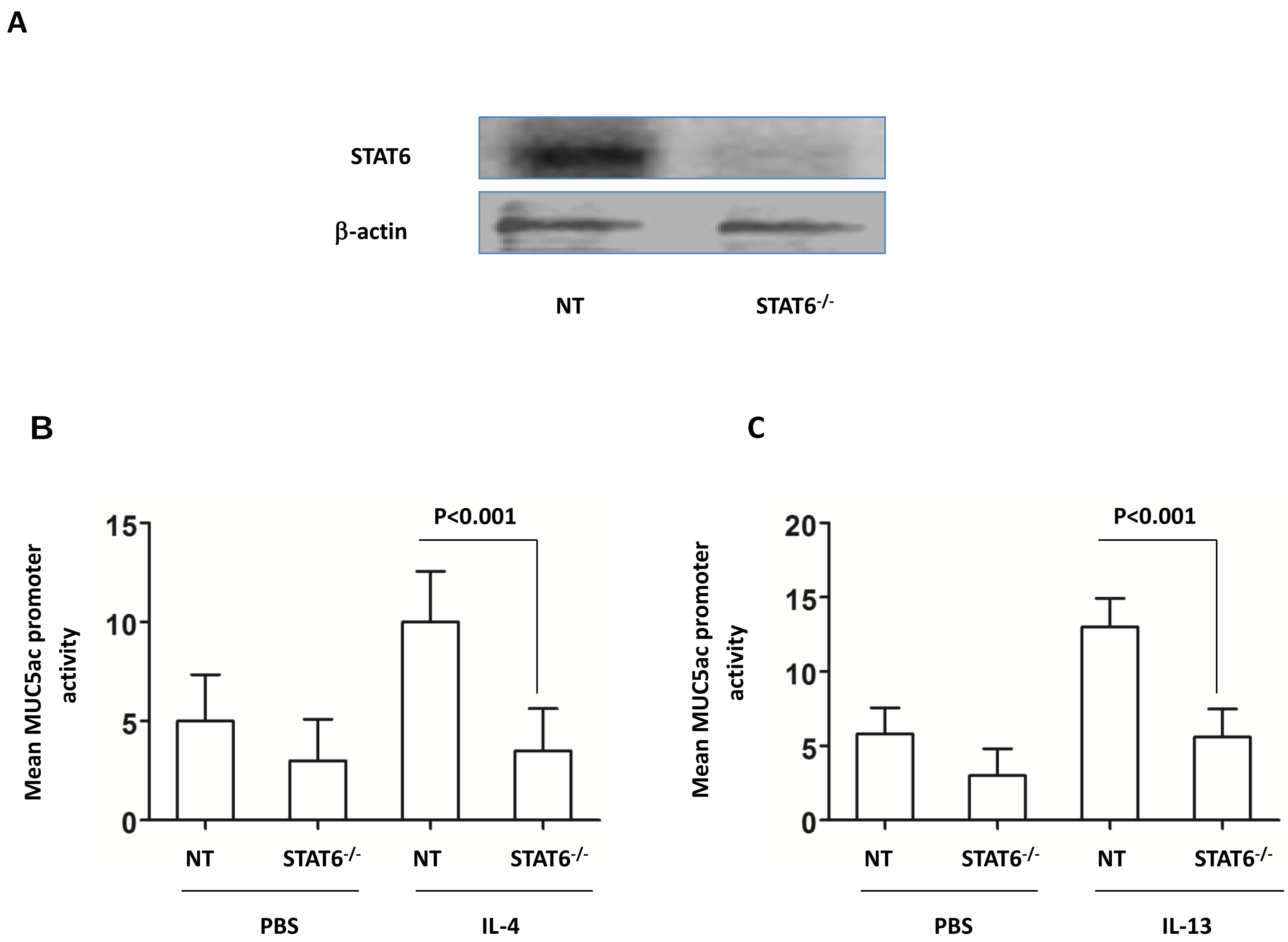

Sup Figure S3

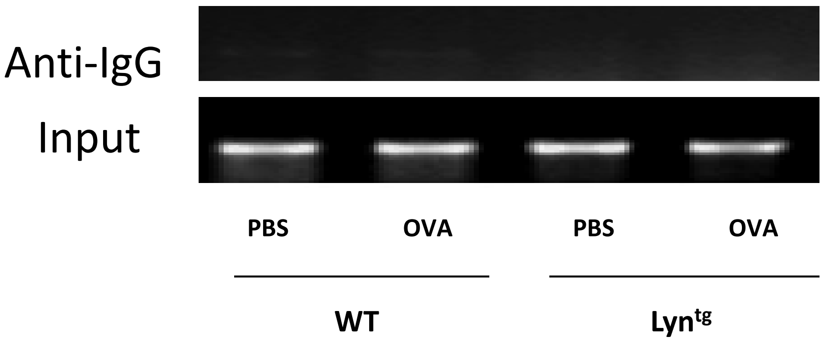

Sup Figure S4

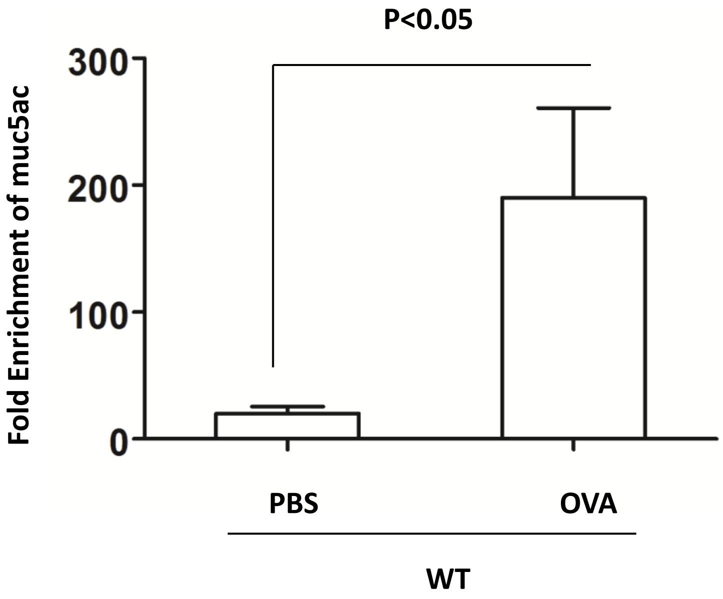

Sup Figure S5
